# Systematic literature review on audio-visual multimodal input in listening comprehension

**DOI:** 10.3389/fpsyg.2022.980133

**Published:** 2022-09-06

**Authors:** Tan Shaojie, Arshad Abd Samad, Lilliati Ismail

**Affiliations:** ^1^School of English, Anhui International Studies University, Anhui, China; ^2^School of Education, Taylor's University, Selangor, Malaysia; ^3^Faculty of Educational Studies, Universiti Putra Malaysia, Selangor, Malaysia

**Keywords:** systematic literature review (SLR), audiovisual input, multimodality, second language (L2) acquisition, listening comprehension

## Abstract

The purpose of this study is to discuss the effects of audiovisual input on second language acquisition (SLA) and the factors that influence the difficulty of audiovisual learning through a systematic literature review. Prior to this systematic review, in this paper, we searched papers on related topics for the past 10 years from 2012 to 2022, and found 46 journal papers that met the research criteria. They can basically represent the scholarly work in this field. The 46 studies were published in journals indexed in Google Scholar, Eric, Scopus, and Wiley Library. Databases were selected according to a set of inclusion and exclusion criteria. The following conclusions are drawn from the literature review: Audiovisual input can provide more authentic language input and more adequate and richer multimodal cultural and situational contexts, which can better promote learners' understanding of the content and stimulate learners' interest in participating in listening comprehension tasks. The influencing factors of multimodal input on listening difficulty include subtitles, video type, and the relationship between the audio and visual input.

## Introduction

With the development of science and technology, second language (L2) listening teaching is changing from traditional audio teaching to audio-visual teaching, and academic circles have become more and more concerned with related research in the use of audio-visuals (Zhyrun, 2016; Namaziandost and Nasri, 2019; Arbab, 2020). Audio-visual input activates both visual and auditory perceptions while audio input only activates auditory perception (Surguladze et al., 2001; Campbell, 2008) and hence, audio-visual input can be considered as a kind of multi-modal input, which is mainly manifested in the form of image (dynamic), sound, and subtitles, and embodies three meta-functions of image, text, and action. Relevant studies have shown that the efficiency of obtaining information through the combination of audio and visuals is far more effective than through either one of the inputs on its own, and that the information is more durable in the memory (Chao et al., 2015; He et al., 2015). Obviously, the distinction between just listening and listening comprehension is important as listening can be an ability to listen without any interpretation and response while listening comprehension involves the complex process of the brain's selection and processing of information. In this complex listening comprehension process, there are some external factors that interfere in or promote listening comprehension, such as the use of the two different input forms of audio and video.

The importance of examining the effects of audio and visual input, both as a single input and when combined, is due to the increased use of modern technology in the classroom and for varied educational purposes. With the aid of multimedia technology, some large-scale tests, such as TOEFL's iBT including national based examinations such as China's CET-4 and CET-6 internet-based tests, began to use audiovisual input materials such as pictures or videos (Wang et al., 2014). As early as the 1990s, a large number of multimedia materials were used in second/foreign language classrooms, and researchers began to explore the impact of visual teaching materials on learning and learners' psychological cognitive factors. Since then, there has been increasing interest in the use of audiovisual input in listening tests. Research related to this has developed for more than 20 years. However, there is still no conclusive among the findings, especially with respect to how audio-visual input affects second language learning and what elements of this input educators should pay attention to.

In exploring the effect of video (audio and visuals combined) and audio-only on listening comprehension, scholars have made further discoveries through empirical research. Some scholars believe that compared with audio-only materials of the same content, audio and visual combined materials can reduce the difficulty of listening (Seeber et al., 2010; Zhyrun, 2016). Ginther (2002) also found that the use of videos can complement audio information with scene context. Hu and Zhang (2013) found through empirical research involving Chinese speaking students that the multimodal combination of audio and video with English subtitles has the greatest effect on promoting students' listening content comprehension, followed by audio and video with Chinese subtitles, and audio and video without subtitles, while audio alone has the least effect. Although the video will distract students' attention to a certain extent and cause a “split” effect, with the cooperation of the target language, it can resist the interference of the video to a certain extent, which is beneficial to students' listening comprehension (Cohen, 2014). Some differing voices were also found in the study which showed that excessive or irrelevant or mismatched audio-visual information may interfere with the audio-visual comprehension process (Canning-Wilson and Wallace, 2000).

The theoretical basis of L2 audio-visual multimodal input mainly includes the Input Hypothesis (Gregg and Krashen, 1986) and Cognitive Load theory (Sweller, 2010). Based on these two theories, this study attempts to retrieve and sort out the related research results of L2 audio-visual multimodal input, and then make a systematic literature review on this basis focusing on audio-visual materials in listening comprehension. As will be discussed in the next section, much research on audio-visual input in language learning conducted prior to the last decade have focused on two central concerns. The first is research on the influence of audio-visual multimodal input on second language acquisition, and the other is the research on the factors that affect the difficulty of second language audio-visual multimodal input.

From the perspective of conducting a systematic literature review, the research questions play a critical role in determining the search strategy, data extraction, and analysis. The research questions identified in this study are given below:

1. What are the effects of audiovisual multimodal input on second language listening comprehension?
2. What are the key factors that affect listening comprehension performance when using visual input?

This paper starts with the introduction to the study, followed by the influencing factors of audio-visual multimodal input difficulty, then, the research protocol and the execution of the systematic literature review are described. This is followed by the findings and discussion of this study. Finally, the conclusion of the study is presented.

## Influencing factors of audio-visual multimodal input difficulty

Based on the above research results, since the 1990s, researchers have carried out empirical studies to examine the factors affecting the difficulty of multimodal input of second language audio-visual, among which three factors have received more attention: audiovisual input, text type, personal factors (Bloomfield et al., 2010; Peters and Muñoz, 2020).

### Visual input

Existing listening tests can use five different information input methods—audio-only input and four visual inputs, namely context-only still images, context-only video, content still images, and content video (Ockey, 2007). Ockey proposed that with the different input methods of information in the listening test, the way test-takers process information will also change, which will lead to differences in test performance and thus affect the construct validity of the listening test. Therefore, most of the existing research focuses on comparing the impact of different audiovisual input methods on test scores and the performance of test-takers (Ockey, 2007). Earlier studies mainly compared the effect of audio-only input and video recording.

Among the studies that examine the effect of different audiovisual input methods, Rajabi et al. (2021) found in a study involving 91 second language learners that there was no significant difference in student achievement between audio-only and video-mediated exams. He also found that some students, apparently distracted by visual input, chose not to look at the screen.

Coniam (2001) compared the difference between audio-only input and video input with 104 Hong Kong English learners using open-ended test questions. The results showed that the audio-only group performed better than the video group, but the difference was not statistically significant. Moreover, the subjects in the video group did not think that using video as a medium in listening was helpful for listening comprehension, nor did the audio group think that using audio was more beneficial. Conversely, 36% of test-takers reported not looking at the screen at all during the test, and a small number of test-takers found the video to be distracting. Cubilo and Winke (2013) used writing and Note-taking tasks to measure listening comprehension and found that the quality of writing after listening was the same under the conditions of visual and auditory input, but the subjects' note-taking behavior was different—the quality of note-taking involving visual input decreased significantly. In contrast to these findings, however, Wagner (2010) found that the video group performed 6.5% higher on the post-test than the audio group, and the difference was significant. He believes that the reason is that the non-verbal information in the video helps the subjects to improve their performance.

There are also studies comparing the effect of different visual input modalities. Ockey (2007), for example, compared the different performances of the subjects when the listening test used a series of still images that provided only the context and only videos of the context. He observed six college students whose native language was not English, and collected data by means of retrospective reports, interviews, and video recordings, and found that in these two different input presentation methods, the subjects' involvement in visual input was manipulated as the time when the subject's eyes were in contact with the display screen. Under the still-picture condition, the subjects had little involvement with the visual input and responded consistently. Most of the subjects believed that still images were only useful in the initial context of listening comprehension and did not help much afterward, but also did not interfere. However, under the video recording condition, there were strong individual differences in the way participants were involved in visual information. Some subjects thought video recording was very helpful for listening comprehension, while others thought video recording was very disruptive to listening comprehension.

Additionally, studies have explored learners' preference for visual input and its relationship to test performance. Cheng and Chau (2016) used a questionnaire to examine Japanese English learners' attitudes toward video-based listening tests and found that 91.9% of students preferred video-based listening tests compared to audio-only tests. However, the study did not answer whether students who prefer video-based tests benefit from visual aids and achieve better test scores.

Suvorov's (2009) research revolves around this problem but has not found a conclusive answer. Overall, test takers had different preferences for different input methods, but their preference for a particular input method did not significantly improve test scores. For example, some candidates are more comfortable with video input than audio-only input, but their performance on the video-input part of the listening test is not necessarily better than the audio-input part. Interestingly, however, students who preferred video input scored significantly higher on the audio listening section than the video section.

### Text type

The second factor influencing the difficulty of audiovisual multimodal input is text type. Ginther (2002), for example, compared the effects of different types of visual input on TOEFL listening comprehension and found that there was an interaction between text types and visual input types. In his research, Ginther combined visual input with text types. He used context-only visual input (a still photo with the speaker and scene) for two-person conversations, short conversations, and academic discussion sections, and a series of still photos and content visual input for mini-talk sections, including photographs, diagrams, and/or diagrams related to listening input. He found that the content visual input accompanying small dialogues and the situational visual input accompanying academic discussions were helpful for listening comprehension, while the situational visual input in small dialogues slightly hindered comprehension.

Wagner (2007) examined the same issue but focused on the influence of text type on the way subjects process visual input and compares the time spent watching the monitor screen of 36 subjects when academic lectures and dialogue videos are played in the listening test. Overall, subjects looked at the monitor screen 69% of the time when the video was being played but watched the dialogue (72%) longer than the academic lecture (67%). Wagner believes that the reason is that dialogue is the interaction between two speakers, with a high degree of contextual dependence, so the contextual cues and non-verbal information are numerous and significant, which are very helpful for learners' understanding. In contrast, lectures are less context-dependent, less interactive, and non-verbal information is poor and unclear. Suvorov's results are consistent with this. He found that the use of video in dialogue had little effect on students' listening comprehension, but the use of video in lectures hindered comprehension (Suvorov, 2009).

### Personal factors

The third factor influencing the difficulty of audiovisual multimodal input is personal factors. Currently, studies that specifically examine personal factors in visual input processing are rare. However, the results of many studies have incidentally found that the personal factors of the subjects, such as learning style, cultural background, language level, etc., may affect their performance in the listening test with visual input (Ockey, 2007; Rajabi et al., 2021).

In a study by Fay and Buchweitz (2014), the hypothesis of the study was that personal factors in working memory capacity of L2 learners would predict listening comprehension performance in a proficiency test. The experiment was conducted in two stages, and the participants included 24 students. In the first part of the experiment, 24 students were given a listening test. In the second part of the experiment, 24 students were tested for working memory span. The experimental results show that larger the working memory storage capacity is, the higher the scores in listening comprehension tasks will be.

A study by Masrai (2020) was conducted among 130 non-native English speakers and examined how much of the differences in listening comprehension were explained by auditory vocabulary knowledge, written vocabulary knowledge, and working memory capacity. Results showed that auditory vocabulary knowledge was the strongest predictor of listening comprehension, followed by working memory ability, while written vocabulary knowledge contributed little. This study discusses the influence of auditory vocabulary knowledge and working memory on the interpretive power of listening comprehension and teaching practice in the second language classroom.

To sum up, existing research has proved that visual input has an impact on the performance of second language learners on listening tests, but whether the impact is positive or negative, and the extent of the impact is still inconclusive. Research has also begun to focus on the interaction between visual input and other factors, such as text type and personal factors, but such research identifying influential factors in this interaction is still in its infancy.

## Systematic review protocol

**Figure 1:**
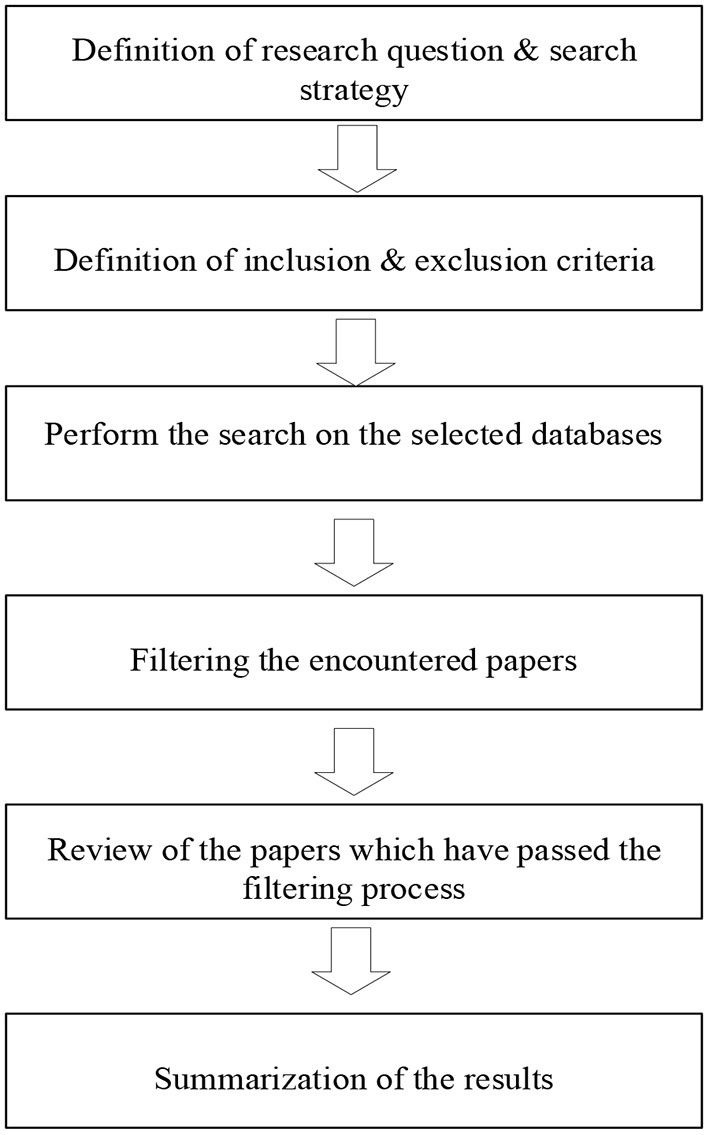
Steps of SLR in this study.

This section outlines the research methodology and research process as well as the screening criteria in the literature review. This research mainly focuses on the influence of audio-visual multimodal input on second language acquisition and the influencing factors of the difficulty of second language audio-visual multimodal input. Figure 1 illustrates the systematic literature review process used in this study.

The systematic literature review is launched before March 2022, and the collected papers are also the literature from the 10-year period back to 2012 before that. We plan to begin our review by formulating research questions, defining a search strategy, and keywords for search. When defining our search strategy, we will also define inclusion and exclusion criteria, which will tell us what types of studies we should include in our study. We will then perform our search in the database to obtain relevant studies based on our keywords. The keywords will typically result in a set of papers that may or may not be relevant to our study, so we will need to narrow the list by filtering only those studies that may be useful or relevant to our study. Afterward, we will start reviewing these papers and summarize the results based on the analysis performed. The following topics describe these steps in more detail.

### Search strategy

In this section, the approach used for finding the relevant studies to answer the research questions is presented.

#### Data source

All the retrieved journals are from the following databases: Google Scholar, Eric, Scopus, and Wiley Library. It is not easy to find the literature among the many pieces of literature. Here, the keyword index is mainly used to find relevant documents, the search scope is also expanded through the replacement of synonyms, and the secondary search is carried out through the relevant documents.

#### Search terms

The keyword search method to search for relevant literature was used. The keywords were generally selected from the title and abstract and are related to the research objectives of the paper. In the advanced search, two to three keywords were selected at the same time to search side by side, because the focus of each database may be different. For this article, the following search terms were used in performing data searches: audiovisual input, or video input, multimodal listening, video-based, visual-based.

#### Search procedure

**Figure 2:**
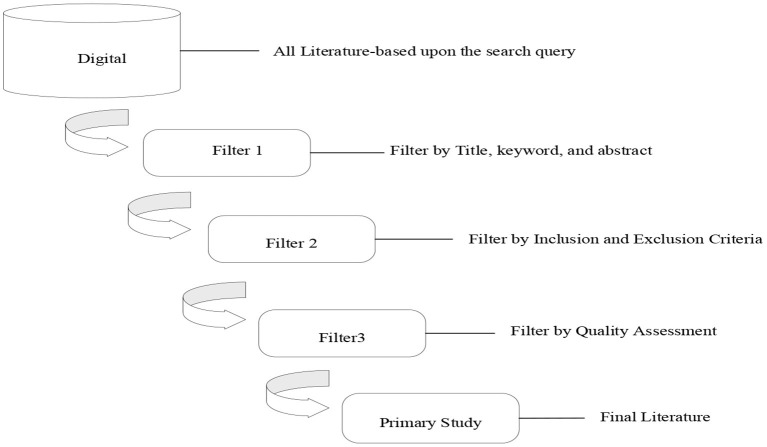
Search process.

Firstly, the data related to this research topic was searched in the paper database, and all papers were screened in three stages. Filter 1 was based on title and abstract keywords of published papers, and studies unrelated to research are excluded. To further refine the results, filter 2 was used, excluding some irrelevant literature. Finally, the rest was filtered for primary studies based on overall quality, using filter 3. For the search procedure, we followed the guidelines provided by Kitchenham et al. (2009) and the entire search process is summarized in Figure 2.

### Study selection

#### Inclusion criteria

The study was selected to find a paper that was relevant to the research question. A key criterion was that the study must focus on the topic of English listening.

The following inclusion criteria were developed:

1. The study had to be a research paper that had been published in peer-reviewed journals and conferences.
2. This was based primarily on the databases Google Scholar, Eric, Scopus, and Wiley online library.
3. The study must be relevant to the research question.
4. The research should be available in open access and full-text format.
5. The research should be published between 2012 and 2022.

#### Exclusion criteria

The following are the exclusion criteria:

1. Studies not written in English.
2. Papers, reports, books. Studies that are not defined as reliable (e.g. web pages).
3. Studies that are not related to our research.
4. Studies that are not accessible.
5. Those studies that are duplicates.

### Quality assessment

Quality assessment (QA) of reviewed literature is paramount to a Systematic Literature Review (SLR) as the quality of conclusions completely depends on the quality of selected literature. Quality Assessment assessing the quality of evidence contained within a systematic review is as important as analyzing the data within. Results from a poorly conducted study can be skewed by biases from the research methodology and should be interpreted with caution. Selecting an appropriate tool to help analyze strength of evidence and imbedded biases within each paper is also essential. If using a systematic review manuscript development tool (e.g., RevMan), a checklist may be built into the software. Other software (e.g., Rayyan) may help with screening search results and discarding irrelevant studies. The following tools may help with study assessment and critical appraisal. The Table 1 below is the specific content of this quality assessment review. The quality assessment team consists of five professors, all of whom are language experts from colleges and universities in China. They will be trained before the review to let them understand the quality assessment standards. There are four options for the standard, [ ] Yes [ ] No [ ] Can't tell [ ] N/A. The selected papers to be reviewed must all meet the review conditions before they can be included in the category of systematic literature review. Only papers with all options of YES can be used. This evaluation team spent two weeks to finally screen out 46 materials from 169 papers that meet the review criteria and can be included in the systematic literature review.

**Table 1:** Criteria used in quality assessment of systematic reviews.

1. Is a focused multimodal listening clearly stated?	[ ] Yes [ ] No [ ] Can't tell [ ] N/A
2. Are the search methods used to identify relevant studies clearly described?	[ ] Yes [ ] No [ ] Can't tell [ ] N/A
3. Was a comprehensive literature search performed?	[ ] Yes [ ] No [ ] Can't tell [ ] N/A
4. Was selection bias avoided?	[ ]Yes [ ] No [ ] Can't tell [ ] N/A
5. Was there duplicate study selection and data extraction?	[ ]Yes [ ] No [ ] Can't tell [ ] N/A
6. Were the characteristics of the included studies provided?	[ ]Yes [ ] No [ ] Can't tell [ ] N/A
7. Was the scientific quality of the included studies assessed and documented?	[ ] Yes [ ] No [ ] Can't tell [ ] N/A
8. Were the methods used to combine the findings of studies appropriate?	[ ] Yes [ ] No [ ] Can't tell [ ] N/A
9. Was the scientific quality of the included studies used appropriately in formulating conclusions?	[ ] Yes [ ] No [ ] Can't tell [ ] N/A
10. Was publication bias assessed?	[ ] Yes [ ] No [ ] Can't tell [ ] N/A
11. Was the conflict of interest stated?	[ ] Yes [ ] No [ ] Can't tell [ ] N/A
12. Are the stated conclusions supported by the data presented?	[ ] Yes [ ] No [ ] Can't tell [ ] N/A

### Data extraction

**Figure 3:**
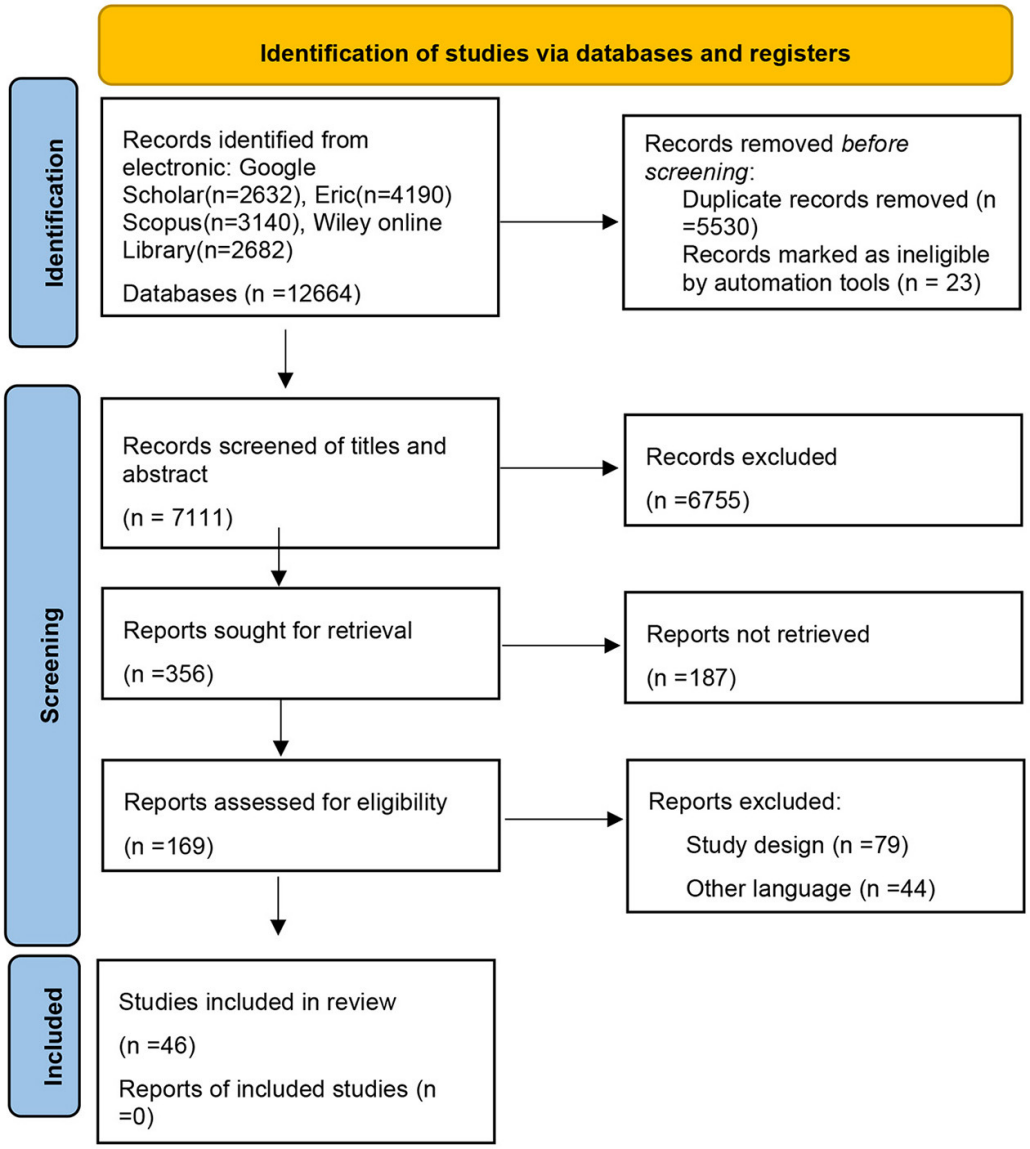
PRISMA 2020 flow diagram for systematic literature reviews.

From the keywords search criteria, a total of 12,664 articles were retrieved. After checking the title and abstract, 12,308 papers that did not match the theme were deleted, and leaving only 356 papers. After considering the inclusion and exclusion criteria, the search procedure was further narrowed to 169 papers, and the final papers were evaluated after the quality assessment of the papers. The literature scope was determined to be 46 articles. The PRISMA 2020 flow diagram for systematic literature reviews is shown in Figure 3 below.

In order to extract the required data for further systematic literature analysis studies, the detailed research content and research gaps of each study were assessed. Literature selected in this paper were collected and searched on related topics from the decade 2012 to 2022. They were preliminarily classified by topic then recorded in the excel sheet, and the items include search engine, item type, publication year, author, title, abstract, research significance, research object, research question, research limitation, research results, etc. Overall Search Result According to the Search Engine are shown in Table 2 below.

**Table 2:** Overall search result according to the search engine.

**No**	**Search engine**	**No. of Initial result**	**Filter1**	**Filter2**	**Filter3**
1	Google Scholar	2,632	89	45	20
2	Eric	4,190	59	22	9
3	Scopus	3,140	129	56	15
4	Wiley online Library	2,682	79	46	2
	Total	12,664	356	169	46

## Findings and discussion

### What are the effects of audiovisual multimodal input on second language listening comprehension?

With the continuous emphasis on multimedia teaching reform, the research on embedding video teaching in listening courses has been increasing in the past few years, and the benefits of video teaching have become increasingly unified. Studies have shown that there is a “compensatory mechanism” in listening strategies (Field, 2004), that is when learners are hindered in listening comprehension due to insufficient language knowledge, they often resort to some compensatory information, such as pictures, videos, and text annotations that can be referred to as well as relevant clues such as cultural information, encyclopedic knowledge, and common sense of life that can be extracted from the listener's own mind. Video, as a compensation mechanism in listening strategy, confirms the possibility and rationality of audio-visual integration from different angle. In fact, compared with pure listening, audio-visual can promote second language learners to use the top-down listening comprehension mode more to make up for the lack of language knowledge (Mohsen, 2016; Pardo-Ballester, 2016). Moreover, compared with pure audio, audio-visual texts can provide more authentic and vivid language input and more adequate and richer multimodal cultural and situational contexts, which can better promote learners' understanding of the content and have a better understanding of the content (Batty, 2015; Lesnov, 2017; Hsieh, 2020).

In terms of the difficulty of listening, some of the research show that audio-visual materials are less difficult than pure audio materials of the same content, and video has a greater role in promoting understanding than audio; audio-visual input can improve the second language learners' understanding of the material text and can promote the development of listening skills. For example, Jaqueline (2019) found that students who trained listening through video stories made faster progress than those who didn't have visual aids. Ockey and Wagner (2018) found that in foreign language learning, the listening comprehension ability of classes with videotaped instructional materials under the guidance of teachers was significantly higher than that of traditional teaching methods. However, in the process of listening comprehension, the use of multimodal input methods needs to consider the connection between cognitive limitation and working memory (Batty, 2021). It is also true that learners are able to process information from multiple sources simultaneously, and when multimodal inputs are properly integrated, learning is most often beneficial (Rogowsky et al., 2016; Bozorgian and Alamdari, 2018). Hence, adding visual modality information to listening comprehension tasks can not only train listeners to increase the capacity of working memory by simultaneously activating audio-visual channels but also help focus their attention to what is important.

### What are the key factors that affect listening test scores when using visual input?

#### The use of subtitles

In visual input, Bairstow and Lavaur (2012) pointed out that subtitles are an important feature of video and important content that affects comprehension. However, They do not discuss the influence of subtitles and subtitle types on audiovisual difficulty but focus on the influence of subtitles on audiovisual understanding. As far as discourse comprehension is concerned, the effect of subtitled audiovisuals is better than that of pure audiovisual. Orero et al. (2018) investigated and compared the effects of three kinds of subtitles on learners' understanding of video content, and found that the subjects who watched the native language subtitles had a better comprehension of the video content than those who watched the target language subtitles (Karakas and Sariçoban, 2012; Winke et al., 2013; Birulés-Muntané and Soto-Faraco, 2016; Ebrahimi and Bazaee, 2016). The test group with target language subtitles was better than the test group without subtitle assistance (Karakas and Sariçoban, 2012; Alabsi, 2020).

After investigating the influence of multimodal input on the listening comprehension of English majors, Lin (2016) found that the multimodal combination of video with English subtitles promoted the students' listening content comprehension the most, followed by pure audio, and the effect of the modality of video with Chinese subtitles are minimal. Lesnov (2022) found that keyword subtitles promote audio-visual comprehension and enhance vocabulary acquisition more than full subtitles. Scholars basically agree that the existence of subtitles can promote audio-visual understanding, but we cannot infer that the existence of subtitles reduces the difficulty of audio-visual (Zhyrun, 2016; Bougiatiotis and Giannakopoulos, 2018). Perhaps as pointed out by (Napikul et al., 2018; p. 158), “reading subtitles may interfere with listening comprehension.” The learner's audio-visual comprehension is likely to be enhanced by reading subtitles, not by audio-visuals alone. Nevertheless, the use of subtitles can be considered a key factor in listening comprehension but the actual influence of subtitles on audio-visual difficulty needs to be further studied (Leveridge and Yang, 2014; Hsieh, 2020).

#### Nature of the visual input

Different types of video images have different effects on learners' second language learning (Gilakjani, 2012; Al Mamun, 2014; Zhang et al., 2017; Winarto et al., 2020). Research so far has largely focused on the impact of two types of video images on visual input: one is content visuals, that is, videos that contain salient information images; the other is context-only visuals that only display the speakers' image exemplified in videos such as talk shows and newscasters reading the news. The content video provides a large amount of information input such as pictures, objects, and real scenes (Dehghani and Jowkar, 2012; Pardo-Ballester, 2016; Green, 2017). Compared with context video, it is seen by some to significantly improve the overall understanding level of learners; but it does not help learners understand the uncommon words they listen to and may even interfere with phonological and vocabulary memory (Gathercole and Baddeley, 2014; Wen et al., 2015).

Gabeur et al. (2020) also argues that the content video input provides more information that is beneficial to listeners' understanding to a certain extent because the scene video only presents the image of the speaker or a fixed scene, the learning process is relatively rigid. Some studies have shown that the close-up of the speaker's head (talking head) can hardly provide additional information to promote the listener's understanding (Crook and Schofield, 2017; Hamdan and Al-Hawamdeh, 2018; Zheng and Samuel, 2019). On the contrary, it may also be possible that the listener at this time tends to focus on pure listening interpretation. Fussalam et al. (2019), for example, found that there was no significant difference in the understanding of the content between the audio-visual and pure listening of the talk show.

Alwehaibi (2015) studied the video recording of the lecture and the comparative input effect of the lecture recording and found that the video group that could see the speaker's facial expressions and body movements had significantly higher scores than the lecture recording group. The same findings have been similarly expressed in studies by other researchers: the learners' comprehension of the lecture content was significantly higher than that of the recording group (Missildine et al., 2013). Both the lecture video and the talk show only presented the image of the speaker, but compared with the pure audio, the experimental results are not consistent (Friesen, 2014; Che et al., 2017). It may be related to the text content of the video in these scenes and the purpose of the video playback. Although the effect of context video in promoting understanding is weaker than that of content video, on the whole, scene video can promote the understanding of language input more than pure audio. Some research shows that video type (news vs. speech) has a significant effect on difficulty perception and audiovisual comprehension (Perez et al., 2014).

#### Relationship between the audio and visual input

Different types of audio and visual relationships in videos will affect audio-visual comprehension, which in turn affects the judgment of the difficulty of audio-visual material (Mathisen, 2012). A direct audio-visual relationship (meaning that the image and voice explanation have a high degree of semantic relevance) and an indirect audio-visual relationship (partial semantic redundancy between the image and the voice explanation) can promote learners' understanding of listening content; however, the close-up of the speaker's head and the discrete sound and picture relationship not only cannot improve comprehension but can even hinder comprehension.

Wong et al. (2012) pointed out that no matter how the audio-visual relationship is coordinated, the information of the visual modality will more or less interfere with the learners. Therefore, to some extent, the relationship between sound and picture may be a cognitive load for learners (Kalyuga and Sweller, 2014). As pointed out earlier, when learners are faced with a more rigid scene video picture, in order to adjust the cognitive load, they are more inclined to only begin interpreting the input through pure “audio-only” processing to obtain information.

To sum up, we found that although there is abundant research on the influencing factors of audio-visual difficulty, there are still some influencing factors to be further proved, especially the further discussion of audio-visual characteristic factors (Akhtar, and Falk, 2017).

## Conclusion

The research on second language audio-visual multimodal input is mainly based on the input hypothesis and cognitive load theory and has a deep understanding of the influence of audio-visual multimodal input on second language acquisition and the factors affecting the difficulty of second language audio-visual multimodal input. In general, compared with traditional single-modal input, audio-visual multimodal input has significant advantages in second language listening comprehension and second language vocabulary acquisition, but what are the advantages of audio-visual multimodal input and how the so-called “multi-modality” should be configured and other issues need further study. SLR research shows that there are some factors affecting audio-visual multimodal input difficulty which are focus on subtitles, different video input, and the relationship between sound and picture, but the influence of language and auditory factors on listening difficulty needs further research. We believe that although the research on L2 audio-visual multimodal input has made great progress, there is still a lot of room for expansion. Grading urgently needs a relatively scientific standard. However, we also believe that with the emphasis on audio-visual multi-modal input and more in-depth exploration in the academic and industrial circles, audio-visual multi-modal input will become the main learning method in second language learning in the future. Moreover, due to the gradual increase in the importance of multimodal teaching methods in the field of second language teaching research, the research methods are also more scientific, and the use of empirical research and technology is becoming more and more extensive.

## Data availability statement

The original contributions presented in the study are included in the article/supplementary material, further inquiries can be directed to the corresponding author.

## Author contributions

TS contributed to the conceptualization, investigation, and writing—original draft. AS and LI contributed to the conceptualization, writing—review and editing, and supervision. All authors contributed to the article and approved the submitted version.

## Conflict of interest

The authors declare that the research was conducted in the absence of any commercial or financial relationships that could be construed as a potential conflict of interest.

## Publisher's note

All claims expressed in this article are solely those of the authors and do not necessarily represent those of their affiliated organizations, or those of the publisher, the editors and the reviewers. Any product that may be evaluated in this article, or claim that may be made by its manufacturer, is not guaranteed or endorsed by the publisher.
